# Model-based process design for surfactin production with *Bacillus subtilis*

**DOI:** 10.1186/s13568-025-01978-3

**Published:** 2025-11-21

**Authors:** Eric Hiller, Manuel Off, Holger Dittmann, Elvio Henrique Benatto Perino, Lars Lilge, Rudolf Hausmann

**Affiliations:** Department of Bioprocess Engineering, Institute of Food Science and Biotechnology, University of Hohenheim, Stuttgart, Germany

**Keywords:** *Bacillus subtilis*, Surfactin, Bioreactor, Bioprocess engineering, Kinetic modeling, High-cell density

## Abstract

*Bacillus subtilis* is one of the most important production organisms in industrial biotechnology. However, there is still limited knowledge about the kinetics of fed-batch processes in bioreactors, as well as a lack of biological performance indicators, such as production yields, particularly regarding their variation over time. Understanding these kinetics and changes is crucial for optimizing the productivity in fed-batch processes. Fed-batch bioreactor cultures of *Bacillus subtilis* BMV9 in high cell density processes for surfactin production have been characterized with a kinetic model composed of first-order ordinary differential equations, describing the time course of biomass, substrate, surfactin and acetate. This model contributes to understanding critical restrictions and the knowledge gained was used to design and implement a model-based process. The model integrates biomass growth based on Monod kinetics, substrate consumption, surfactin synthesis and formation of the by-product acetate. After the model was parameterized for *B. subtilis* BMV9 using 12 different fed-batch bioreactor experiments, the kinetic model was able to accurately describe biomass accumulation, substrate consumption, product formation rates and, to some extent, the overflow metabolism involving acetate. Based on this, the kinetic model was used for a process design, in which the batch was omitted, which led to a product titre of 46.33 g/L and a space–time-yield of 2.11 g/(L*h) was achieved. The kinetic model developed in this study enables the description of the time course of biomass growth, substrate consumption and product formation and thus significantly improves process understanding. The computation of process parameters, which are not analytically accessible at any time, could be realized. A sensitivity analysis identified the maximum specific growth rate, substrate-related maintenance and the maximum acetate formation rate as key parameters influencing model outputs.

**Supplementary Information:**

The online version contains supplementary material available at 10.1186/s13568-025-01978-3.

## Introduction

*Bacillus subtilis* is a well-characterized gram-positive bacterium, well-known for its ability to produce a variety of secondary metabolites, including the potent biosurfactant surfactin (Wenzel et al. 2011; Bonmatin et al. 2003). As a member of bioactive lipopeptides, surfactin is comprised of a cyclic peptide moiety formed by the seven amino acids L-Glu, L-Leu, D-Leu, L-Val, L-Asp, D-Leu and L-Leu. This peptide structure is cyclized by a lactone bond to a fatty acid chain, ranging from 12 to 19 carbon atoms. Due to this complex molecule structure, surfactin has attracted significant attention as a biosurfactant due to its pronounced capability to reduce surface tension as well as its reported antimicrobial properties and potential applications across various areas such as bioremediation, microbially enhanced oil recovery (MEOR) and even in pharmaceuticals (Cooper et al. 1981; Mulligan 2005; Liu et al. 2015; Vollenbroich et al. 1997).

In the context of surfactin production, batch processes are less favorable, compared to fed-batch setups (Bouassida et al. 2023). Among the glucose feeding strategies, namely pulsed, constant and exponential, the latter was proved to be the most effective setups (Bouassida et al. 2023). Due to its lack of sporulation, the surfactin-producing *B. subtilis* strain JABs32 demonstrated a 1.6-fold increase in growth rate when cultivated in shake flasks, making this strain particularly suitable for high cell density fed-batch fermentation processes (Klausmann et al. 2021a). In bioreactor cultures utilizing an optimized mineral salt medium, this strain achieved biomass concentrations of 41.3 g/L and surfactin titres of 23.7 g/L after 31 h of cultivation, highlighting its potential for efficient large-scale surfactin production with an exponential feeding (Klausmann et al. 2021a). Another derivative surfactin-froming and non-sporulating strain from 3NA, called *B. subtilis* BMV9, was cultivated in various fed-batch bioreactor processes with chemically defined mineral salt medium and glucose as carbon source (Vahidinasab et al. 2020). By varying the exponential feeding rates, the strain BMV9 achieved cell dry weights ranging from 35.96 to 49.19 g/L, while producing surfactin in the range of 19.19 to 36.75 g/L (Hiller et al. 2024).

The growth rate of *B. subtilis* is a critical determinant of surfactin production, as it is closely linked to the metabolic state of the cells (Amin 2014). Studies have demonstrated that surfactin production is often coupled with biomass formation under certain conditions. However, fed-batch bioreactor processes with high feeding growth rates also generate by-products such as acetate, due to overflow metabolism, which can have a negative impact on growth and product yield (Hiller et al. 2024; Amin 2014). Understanding this relationship in fed-batch processes is essential to optimize feeding and maximize surfactin production. In addition, further feeding strategies allow the development of completely new process designs (Hiller et al. 2024). However, despite the significant advances in learning about the production of different products, current models have largely focused on batch processes, with limited exploration of fed-batch strategies, particularly those incorporating dynamic kinetic modeling. In this context, kinetic models provide insights into the dynamics of microbial growth, substrate consumption and product formation. These models are valuable tools for prediction and evaluation of key process parameters (Dong et al. 2015). In literature, several kinetic models are available for the description of bioprocesses for the production of various bioproducts. In the field of biosurfactants, a kinetic model incorporating quorum sensing mechanisms has already been developed using the example of rhamnolipids in a batch process (Henkel et al. 2014). Another attempt to mathematically describe the production of biosurfactants in an anaerobic batch process, using an unstructured kinetic model, was made by Alvarado et al. (2022) by analyzing various kinetics, such as the Contois kinetics. In this case, *B. subtilis* was used as the production organism and crude oil was the substrate. In another study, the production of the mycosubtilin lipopeptide was simulated with an exponential feeding process and continuous purification using a dynamic model based on Monod kinetics (Guez et al. 2007). Therefore, the biomass was modeled in both the cultivation medium in the bioreactor and in the foam phase, as foaming was induced with a foam collector. In addition, surfactin was identified as another product, which was localized only in the foam phase. Moshtag et al. (2022) have modeled biosurfactant production with *B. subtilis* in shake flask cultivation as a batch process using brewery waste as a substrate. Heryani and Putra (2017) conducted a study to predict the biomass formation of *Bacillus sp*., biosurfactant formation and surface tension using the modified Gompertz kinetic model in shake flasks. Levisauskas et al. (2024) developed a hybrid mathematical model for the simulation of *Azotobacter vinelandii* growth, biosurfactant formation and substrate consumption using mass balance differential equations in a fed-batch process with a fermenter scale of 5 L. Ee Chuo et al. (2023) simulated the the biosurfactant production in a batch process using crude oil as a substrate and a sludge-isolated bacteria as the producer organism based on the kinetic model of Alvardo et al. (2022).

In contrast to biosurfactants but with the usage of *Bacillus* species, unstructured kinetic models have been successfully applied to the production of polyhydroxybutyrate using *B. subtilis*, resulting in insights into the optimization of fed-batch processes (Yadav and Patra 2023). Furthermore, the production of nattokinase with *B. subtilis* was described using an unstructured kinetic model that represents a batch process (Vinayagam et al. 2015). Similarly, structured models have been utilized in sodium gluconate fermentation processes, using *Aspergillus niger*, highlighting their flexibility and applicability across different bioprocesses (Dong et al. 2015).

Compared to previous studies, this work expands kinetic modeling by explicitly incorporating the effects of overflow metabolism on both cell growth and product formation. Acetate, used as a representative overflow metabolite, was added to the model as an additional state variable, resulting in a system of four first-order ordinary differential equations describing the time course of biomass, substrate, surfactin and acetate. Biomass growth inhibition by acetate was described as a substrate-dependent term, in which high substrate availability leads to increased acetate formation and subsequent inhibition of growth and surfactin formation. In contrast to earlier models, both the batch and the exponential feeding phases were considered to capture time-resolved process behavior. The kinetic model was parametrized using previous experimental data from multiple fed-batch bioreactor experiments conducted at various exponential feeding growth rates (Hiller et al. 2024). The model satisfactorily simulates the influence of exponential feeding rates on biomass formation, surfactin production and the impact on the overflow metabolism, providing a comprehensive understanding of the underlying kinetics and offering valuable insights for the industrial-scale production of surfactin. Additionally, the kinetic model is utilized to develop a new process design and predict process outcomes, including final titre and space–time-yield.

## Materials and methods

### Chemicals and standards

Unless otherwise stated, the chemicals used in this study were purchased from Carl Roth GmbH & Co. KG (Karlsruhe, Germany). Surfactin standard (≥ 98% purity) was purchased from Sigma-Aldrich Laborchemikalien GmbH (Seelze, Germany).

### Bacterial strain, media and conditions for fed-batch and model-based cultivations

In this study, *Bacillus subtilis* strain BMV9 (*spo0A3*; *trp*^+^; *sfp*^+^; Δ*manPA*) was used (Vahidinasab et al. 2020). The media and components used for precultures were already described by Klausmann et al. (2021a). Briefly, the first pre-culture was carried out in LB medium, while a chemically defined mineral salt medium was used for the subsequent second pre-culture. Shake flask cultivations were performed in an incubator shaker (NewbrunswickTM/Innova 44, Eppendorf AG, Hamburg, Germany) at 37 °C and 120 rpm.

Bioreactor cultures were carried out in a 30 L fermenter (ZETA GmbH, Graz/Lieboch, Austria). Experimental data of cell dry weight, glucose, surfactin and acetate were used for parametrization of the kinetic model. Overall, data from fed-batch cultivations at different exponential feeding growth rates, namely 0.075, 0.15, 0.2, 0.25, 0.3 and 0.4 1/h were taken from Hiller et al. (2024). The conditions used for the bioreactor experiments were described in Hiller et al. (2024) with some minor changes for the model-based process design experiments: the initial batch volume was reduced to 10 L, consisting of salts, trace elements and magnesium sulphate as described in Klausmann et al. (2021a) excluding batch glucose. The medium was inoculated to an initial OD_600_ of 0.3 and cells were cultured at a constant temperature of 37 °C and pH of 7. The initial aeration rate was set to 5 L/min and was adjusted stepwise up to 72 L/min to keep a pO_2_ value of 50% constant. In addition, the starting point of the exponential feeding phase was set to 1 min after inoculation, as described by Amin (2014) and the feeding volume was increased from 6 to 10 L. The initial feeding rate F_0_ (g/h) for the 50% (w/v) glucose feed solution was determined using the kinetic model and was used to calculate the feeding rate F(t) (g/h) at every time point (t) of the experiment, using the feeding growth rate µ_F_, set to 0.2 1/h (Eq. (1)).

1 $$F\left(t\right)= {F}_{0} *{e}^{{\mu }_{F} *t}$$

To find the optimum initial feeding rate F_0_ for the model-based design, the parameters maximum surfactin titre P_end_ and space–time-yield P_V_ were selected with the goal of maximization. Equation (2) provides the relationship for calculating P_V_.

2 $${P}_{V}= \frac{{P}_{end}}{{V}_{end} * {t}_{end}}$$

In this equation, P_V_ (g/(L*h)) is the space–time-yield, P_end_ (g) is the amount of surfactin when the glucose feed was depleted, V_end_ (L) is the reactor filling volume at the end of the process and t_end_ (h) is the process time.

### Sample analysis

Samples taken during cultivation were centrifuged at 3890 xg for 10 min at 4 °C (Multifuge X3R, Thermo Fisher Scientific, Waltham, USA). The cell-free supernatants were used to quantify acetate and glucose with enzymatic assay kits (R-Biopharm AG, Darmstadt, Germany). The cell dry weight (CDW) was calculated from experimentally determined OD_600_ values with a correlation factor of 0.232 as described by Hiller et al. (2024).

### Surfactin quantification

The amount of surfactin produced during the cultivation process was quantified by high-performance thin-layer chromatography (HPTLC) (CAMAG AG, Muttenz, Switzerland). The exact measurement methods are mentioned in Geissler et al. (2017). For extraction, 2 mL of the centrifuged cell-free supernatant was subjected to a chloroform/methanol mixture (2:1) in three steps. The lower phase was subsequently evaporated at 40 °C and 10 mbar using a rotary evaporator (RVC 2–25 Cdplus, Martin Christ Gefriertrocknungsanlagen GmbH, Osterode am Harz, Germany). The dried residue was dissolved in 2 mL of methanol and applied in 6-mm bands onto a silica HPTLC plate. Plate development was carried out over a migration distance of 60 mm using a mobile phase composed of chloroform/methanol/water (65:25:4). Surfactin detection was performed at 195 nm (Geisler et al. 2017), with a surfactin standard (Sigma Aldrich, Seelze, Germany) for quantification.

### Modeling

#### Modeling platform

Modeling was performed with the mathematical/numerical program MATLAB R2023a (MATLAB, The MathWorks, Natick, MA, USA). The differential equations system was solved using the implicit multi-step solver “ode15s”, which was embedded in the MATLAB environment (Shampine and Reichelt 1997).

#### Nomenclature

The model consists of the biomass X the substrate (glucose) S the product (surfactin) P and a by-product of the overflow mechanism (acetate) A. In addition the volume of the medium within the bioreactor V the feeding volume v and the process time t were implemented. An overview of all model parameters and variables along with their units and definitions is provided in Table S1 in the supplementary material

#### Model setup

##### Initial conditions

The initial values of biomass X_0_, glucose S_0_, surfactin P_0_ and acetate A_0_ were calculated from the raw data at the beginning of each exponential feeding experiment as described by Hiller et al. (2024). For the model-based process, the initial biomass X_0_ was calculated from the starting CDW, while all other initial conditions were set by the pre-culture.

##### Biomass growth

Growth of biomass X (Eq. (3)) was set to be directly proportional to the already existing biomass, the specific growth rates µ_S_ for glucose as substrate and µ_A_ for acetate as an alternative substrate.

3 $$\frac{dX}{dt}= {\mu }_{S} \cdot X+ {\mu }_{A} \cdot X$$

The specific growth rate µ_S_ (Eq. (4)) was expressed by the Monod kinetics (Monod 1949). Here, ${\upmu }_{\text{max}}^{\text{S}}$ means the maximum specific growth rate of biomass X on glucose as substrate S, divided by the filling volume of the bioreactor V, and K_S_ represents the half-saturation constant, which was fixed as reported by Henkel et al. (2014). The Monod kinetics were extended by a general inhibition term, which describes the inhibition of growth by acetate A, with the inhibition constant K_I_ (Yano and Koga 1973).

4 $$ \mu_{S} = \mu_{max}^{S} \cdot \frac{\frac{S}{V}}{{\frac{S}{V} + K_{S} }} \cdot \frac{{K_{I} }}{{\frac{A}{V} + K_{I} }} $$

The growth rate of biomass X on acetate A was expressed by the Monod kinetics (Monod 1949) as well (Eq. (5)). The specific growth rate µ_A_ was calculated using the maximum specific growth rate ${\upmu }_{\text{max}}^{\text{A}}$ and the half-saturation constant K_A_ which was set fixed equal to K_S_. In order to allow cell growth on acetate, three conditions needed to be fullfilled: acetate must be available, the glucose concentration must be below a critical level c_S,crit_ and glucose must be available which is particularly important because *Bacillus* cannot grow on acetate alone without the presence of another carbon source (Grundy et al. 1993).

5 $$ \mu_{A} = \mu_{max}^{A} \cdot \frac{\frac{A}{V}}{{\frac{A}{V} + K_{A} }} $$

##### Substrate consumption

The consumption of the substrate glucose S is shown in Eq. (6). Glucose was fed into the cultivation system through the feed F. The concentration of glucose in the feed was used to calculate the amount of substrate S added per unit of time. The glucose feed, along with the initial glucose present in the batch, was used for the calculation of the growth of biomass X, the formation of the product of interest, namely surfactin, P and the formation of the by-product, namely acetate, A. Conversion yields Y_X/S_ for biomass, Y_P/S_ for surfactin and Y_A/S_ for acetate were applied for these processes. In case the by-product acetate was used for the growth of biomass, the term $\frac{1}{{Y_{{A/S}} }} \cdot \frac{{dA}}{{dt}}$ was assumed to be zero.

6 $$\frac{dS}{dt}= \frac{F}{{\rho }_{Feed}} \cdot {C}_{S,Feed}- \frac{{\mu }_{S}}{{Y}_{X/S}} \cdot X- \frac{1}{{Y}_{P/S}} \cdot \frac{dP}{dt}- \frac{1}{{Y}_{A/S}} \cdot \frac{dA}{dt}$$

The conversion of glucose to surfactin and acetate was expressed using stoichiometric yields Y_P/S_ and Y_A/S_. However, the conversion factor of glucose to biomass Y_X/S_, described by Pirt (1965), was expressed as a function of the specific growth rate µ_S_ and the constant theoretical maximum conversion yield ${\text{Y}}_{\text{X}/\text{S}}^{\text{true}}$ (Eq. (7)). The equation also took into account the non-growth-associated maintenance requirements with the maintenance coefficient m_S_.

7 $${Y}_{X/S}= \frac{{\mu }_{S}}{\frac{{\mu }_{S}}{{Y}_{X/S}^{true}}+ {m}_{S}}$$

##### Product formation

The formation of the target product P, specifically surfactin, was calculated to be directly proportional to the growth of biomass X with a specific growth rate μ_S_ as described in Eq. (8). The amount of surfactin produced per unit of biomass was expressed using the yield coefficient Y_P/X_.

8 $$\frac{dP}{dt}= {\mu }_{S} \cdot {Y}_{P/X} \cdot X$$

##### Acetate formation

The formation of the by-product acetate A is part of the overflow metabolism in *B. subtilis* (Kabisch et al. 2013). Acetate was selected as a representative for all excess metabolites, as it was assumed that the metabolite is the most relevant in terms of inhibitory effects. Accordingly, it was assumed that the formation of acetate only appears in the presence of a critical glucose concentration ${C}_{S,crit,A1}$. Consequently, further assumptions were an absence of an acetate formation rate b below this concentration. In addition, it was defined that a maximum formation rate b_max_ would be present, when a second critical glucose concentration ${C}_{S,crit,A2}$ is exceeded. In the intermediate range, an acetate formation rate was described with a linear relationship (Eq. (9)).

9 $$b = \frac{{b}_{max}}{{C}_{S,crit,A2}- {C}_{S,crit,A1}} \cdot \frac{S}{V}- \frac{{b}_{max} \cdot {C}_{S,crit,A1}}{{C}_{S,crit,A2}- {C}_{S,crit,A1}}$$

The formation of acetate A by the biomass X was assumed to be directly proportional to the formation rate b. Furthermore, it was predicted that the biomass is able to use the by-product acetate as a carbon source for growth with a specific growth rate µ_A_ and a conversion yield Y_X/A_, as described previously (Hiller et al. 2024). In addition, the maintenance requirement m_A_ was assumed to be another usage of acetate during the bioprocess, as this describes the fractional consumption of acetate for biomass maintenance under conditions of low glucose availability. The value of m_A_ becomes zero when acetate is not utilized for growth. Equation (10) describes the change of acetate over time mathematically.

10 $$\frac{dA}{dt}= b \cdot X- {m}_{A} \cdot X- \frac{{\mu }_{A}}{{Y}_{X/A}} \cdot X$$

### Filling volume of the bioreactor and feeding volume

The filling volume of the bioreactor V increased with the feed rate F, the addition of antifoam, acid and base for pH-control, while the bioreactor volume V decreased by sampling. Volume changes of the liquid due to foaming was considered as insignificant, because of the usage of a foam centrifuge and an antifoaming agent to avoid overfoaming. The volume change due to evaporation was also considered negligible, as the reactor is equipped with an off-gas cooler with recirculation. The volume correction factor c accounts for the average contributions over the 12 fed-batch bioreactor experiments, including the addition of pH-adjusting agents, antifoam solution, sampling, and the water content of the feed. In the batch phase, the filling volume of the bioreactor was assumed to be constant, as no antifoam was used and the loss of volume by sampling was equivalent to the addition of acid and base. Accordingly, no volume correction was needed until the initiation of the feeding phase. To transfer the feed mass flow to a volumetric one, F was divided by the density of the feed ρ_Feed_. Equation (11) describes the change of the filling volume in the bioreactor.

11 $$\frac{dV}{dt}= c \cdot \frac{F}{{\rho }_{Feed}}$$

The feed volume v was predicted to be directly linked to the feed rate F divided by the density of the feed solution ρ_Feed_ (Eq. (12)). The modeling was stopped when the volume of the feed solution was taken and thus dv/dt became zero.

12 $$\frac{dv}{dt}= - \frac{F}{{\rho }_{Feed}}$$

### Exponential feeding

The initial feed rate F_0_ was calculated when the glucose from the batch cultivation dropped below the critical substrate concentration C_S,crit_. At this time point, the model assigned the corresponding values to X_FS_ for the biomass and t_FS_ for the time point during the feeding phase and then calculated the biomass yield for the batch phase Y_X/S,Batch_. Using the additional parameters m for maintenance, the density ρ_Feed_, the feeding growth rate µ_F_ and the glucose concentration in the feed C_S,Feed_, the value of the initial feed rate was calculated according to Eq. (13). For the model-based process design approach, the initial feed rate varied. The feeding rate F at every time point was calculated with Eq. (1) as described before.

13 $${F}_{0} =\left(\frac{{\mu }_{F}}{{Y}_{X/S,Batch}}+{m}_{S}\right) \cdot \frac{{X}_{FS}}{{C}_{S,Feed}} \cdot {\rho }_{Feed}$$

### Statistical evaluation

For the statistical evaluation of the model, the RMSE value was used. This metric shared the same units as the model fits to be evaluated, making differences between experimentally measured and modeled data clearer. Ideally, RMSE values are close to zero, indicating a perfect fit. In addition, RMSE was particularly sensitive to outliers, which means it effectively highlighted large discrepancies between predicted and observed values, further increasing the accuracy of the model evaluation (Chai and Draxler 2014).

### Model validation with carbon mass balance

To validate the kinetic model, a carbon mass balance was performed based on simulated end-point amounts. The calculation included the main carbon-containing components substrate (glucose), biomass, product (surfactin) and acetate. All components were converted to carbon-equivalents (mol C) using molar conversion factors derived from their molecular composition and molar mass. Glucose was converted using a factor of 0.0333 mol C/g, based on its molecular weight of 180.16 g/mol and six carbon atoms per molecule. Biomass was converted using 0.04027 mol C/g, assuming a molar mass of 24.83 g/mol C for *Bacillus subtilis* biomass, as reported by Dauner et al. (2001). Surfactin was converted using 0.0511 mol C/g, based on a molar mass of 1036.34 g/mol and 53 carbon atoms per molecule. Acetate was included using a factor of 0.0333 mol C/g, corresponding to two carbon atoms per molecule and a molar mass of 60.05 g/mol. The carbon recovery was calculated as the ratio of the total carbon equivalents in biomass, product and by-product to the total carbon input via glucose feeding.

### Sensitivity analysis with Morris method

To analyze the sensitivity of the model and figure out the most influential model parameters, a global sensitivity analysis was conducted using the Morris method (Morris 1991). In total 17 relevant model parameters were varied within a range of ± 10% around their respective reference values. The analysis was done using the SAFE (Sensitivity Analysis For Everybody) toolbox (Pianosi et al. 2015). The sampling procedure was based on 50 trajectories, each generated with 6 discretization levels and a step size of 0.2, which ensured an adequate resolution for the parameter space while maintaining numerical stability. To ensure reproducibility, a random seed of 42 was set before sampling. As objective functions, the RMSE between model simulations and experimental data were calculated for biomass, substrate, product and acetate. Separate Morris analyses were performed for each RMSE based objective. The mean absolute elementary effect ${\mu }^{*}$ was expressed as the result (Campolongo et al. 2007), providing a quantitative measure of each parameter`s relative influence on model accuracy.

### Data analysis

In order to evaluate the model-based process design and compare it with other experiments, the production yields, namely product per biomass (Y_P/X_), product per substrate (Y_P/S_), biomass per substrate (Y_X/S_), as well as the specific productivity (q_P/X_) and specific substrate-product conversion rate (q_P/S_) were calculated. The equations used for calculation were taken from Hiller et al. (2024). In addition, the space–time-yield was calculated according to Eq. (2).

### Plotting of experimetal data and model fits

All graphs were generated with OriginPro 2022b (OriginLab Corporation, Northampton, USA) software.

## Results

### Model parametrization

In a first step, differential equations were established on the basis of absolute values that explain cell growth, substrate consumption and the formation of both the target product surfactin as well as the by-product acetate. In addition, exponential feeding equations have been defined. Here, certain parameters were used as fitting parameters, meaning they were adjusted simultaneously during the modeling process to closely match the experimental data according to the RMSE and ensure an accurate simulation of the biological system. To evaluate the impact of acetate on biomass growth, product formation and substrate consumption, a simplified model was implemented in which acetate-related effects were excluded. Specifically, the acetate formation rate b (Eq. (9)) and the corresponding differential equation for acetate (Eq. (10)) were set to zero. Biomass growth on acetate (Eqs. (3) and (5)) was also omitted, along with substrate consumption associated with acetate formation (Eq. (6)). Lastly the inhibition term in the Monod kinetics (Eq. (4)) was removed. The model was subsequently parametrized for a feeding growth rate of 0.4 1/h, a condition under which overflow metabolism is strongly triggered. However, this parametrization led to biologically and physically implausible results, such as negative lag phase. This highlights the critical need to account for overflow metabolism in the modeling process. The parameters, including the fitting ranges, for the bioprocesses with different exponential feeding rates under consideration of acetate are provided in Table 1. An additional Table 2 shows all the parameter changes made for the model-based process design approach.

**Table 1:** Parameters with ranges used for the modeling of the experiments shown in Hiller et al. (2024) and the model-based process design approach. The strain for which these parameters apply is *B. subtilis* BMV9, with general process parameters being 37°C, pH 7 and pO_2_ 50%

Parameter	Value	Unit	Range	Comment/source
*Growth kinetics*				
${\mu }_{max}^{S}$	0.5	1/h	0.36–1	Fitting parameter
${\mu }_{max}^{A}$	0.5	1/h	0.36–1	Fitting parameter
${K}_{S}$	0.05	g/L	–	Fixed glucose affinity
${K}_{A}$	0.05	g/L	–	Fixed acetate affinity
*Yields*				
${Y}_{X/S}^{true}$	0.45	g/g	0.4–0.46	Fitting parameter; Sauer et al. (1996)
${Y}_{P/X}$	0.54–0.75	g/g	0.54–0.75	Hiller et al. (2024)
${Y}_{P/S}$	0.46	g/g	–	Stoichiometry
${Y}_{A/S}$	0.67	g/g	–	Stoichiometry
${Y}_{X/S,Batch}$	0.28	g/g	0.22–0.37	Model estimated
${Y}_{X/A}$	0.45	g/g	–	Fitting parameter
*Maintenance*				
${m}_{S}$	0.05	g/(g*h)	–	Hiller et al. (2024)
${m}_{A}$	0.05	g/(g*h)	–	Same value as ${m}_{S}$ was used
*Lag phase*				
${t}_{Lag}$	0.25–5.25	h	0.25–5.25	Fitting parameter
*Exponential feeding*				
${\mu }_{F}$	0.075–0.4	1/h	0.075–0.4	Experiments from Hiller et al. (2024)
${c}_{S, crit}$	0.05	g/L	0.005–0.5	Fitting parameter
${c}_{S, Feed}$	500	g/L	–	Klausmann et al. (2021a)
${\rho }_{Feed}$	1180	g/L	–	Experimental value
X_FS_	84	g	67–111	Model estimated
t_FS_	–	h	12.5–16	Model estimated
*Volume correction*				
$c$	0.8	–	–	Experimental value
*Acetate formation*				
${b}_{max}$	0.072	1/h	0.01–0.2	Fitting parameter
${c}_{S,crit,A1}$	0.075	g/L	0.05–0.1	Fitting parameter
${c}_{S,crit,A2}$	5	g/L	1–10	Fitting parameter
*Inhibition*				
${K}_{I}$	5	g/L	1–10	Fitting parameter

**Table 2:** Parameter changes made for the model-based process design approach

Parameter	Value	Unit	Range	Comment/source
*Yields*				
${Y}_{P/X}$ Lag phase	0.71	g/g	–	Hiller et al. (2024)
${t}_{Lag}$	0.25	h	–	–
*Exponential feeding*				
${F}_{0}$	28	g/h	1–100	Model result
${\mu }_{F}$	0.2	1/h	–	–

Overall, the model is comprised of 24 parameters and 6 of them being yields (Table 1). Of the total 24 parameters, 5 were derived from stoichiometry or set fixed, 3 were derived from literature, 2 were determined experimentally, 3 were estimated during the modeling process and 11 were used as fitting parameters.

### Exponential feeding rate experiments

**Fig. 1:**
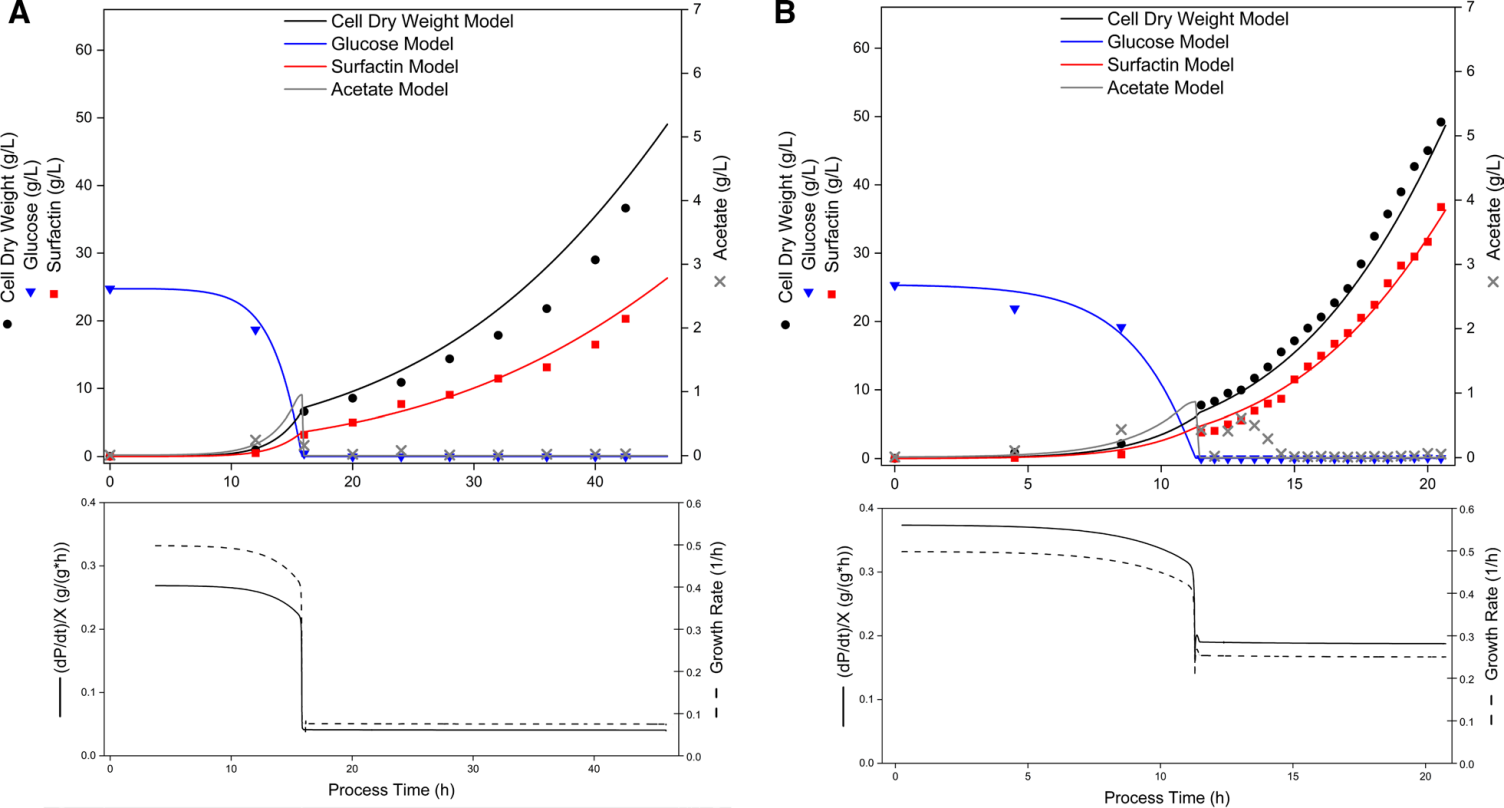
Time course of bioreactor processes for the cultivation of *B. subtilis* BMV9 with two different exponential feeding growth rates 0.075 1/h (**A**) and 0.25 1/h (**B**) and modeling of the data. Measured experimental parameters, taken from Hiller et al. (2024), were cell dry weight (black circles), glucose (blue inverted triangles), surfactin (red squares) and acetate concentration (grey crosses). The lines through the data points of cell dry weight, glucose, surfactin and acetate represent the model fits. In addition, the specific growth rate of biomass (black line) due to the consumption of the substrate glucose and the biomass productivity (black dashed line) were continuously represented below

Experimental process parameters cell dry weight (X), glucose (S), surfactin (P), acetate (A) and reactor filling volume (V) from Hiller et al. (2024) were used to parametrize the model. The data employed, provide duplicate determinations from 12 individual experiments distributed across 6 different exponential feeding growth rates, namely 0.075, 0.15, 0.2, 0.25, 0.3 and 0.4 1/h. The data of all experiments and simulation results are provided in Table S2 in the supplementary material. Figure 1 illustrates an example of the temporal progression of the process parameters for the feeding growth rates 0.075 and 0.25 1/h, along with the corresponding simulated data. In addition, continuous profiles of the specific growth rate of the biomass and the cell productivity related to the production of surfactin were integrated as important biological performance indicators. Before each of the exponential feeding rate experiments could started, a respective initial batch phase was performed. Therefore, a certain amount of cells was added as inoculum to a defined amount of glucose. Once the initial substrate was consumed through cell growth, maintenance and the formation of surfactin as target product as well as overflow metabolism represented by acetate, the fed-batch phase was initiated by starting the glucose feed. The experiment was finished after depletion of the feed volume. By varying the lag phase (Table 1) of the biomass at the beginning of the batch phase, the starting point of the exponential feeding was predicted by the model with an average deviation of 0.076 h. At the start of the feeding phase, the initial feed rate was calculated by the model according to formula (12). This rate determined the end point of the modeling process. The calculation was primarily based on the biomass present at that stage, with an average deviation of 1.09 g/L compared to the measured data, which led to an average deviation in the feed time of 0.93 h. Comparing the process end time between the experimental and modeled values resulted in a mean error of 0.88 h.

The batch phase was consistent across all experiments, only the initial values for biomass, glucose, surfactin, acetate and the duration of the lag phase, in which the cells adapt to the bioreactor environment, varied slightly. In all experiments, glucose decreased due to biomass growth, maintenance and the formation of surfactin and acetate, which was also confirmed by the model. After the lag phase, biomass formation of up to a maximum specific growth rate of 0.5 1/h could be shown in the batch phase, before a decline was observed towards the end of the batch cultivation process. Similarly, the cell productivity reached a peak during the batch phase. The starting of exponential cell growth was evident in both the model and experimental data after the lag phase. The growth-associated production of surfactin also followed the trend in both the modeled and experimental data, with production yields in the range of 0.54–0.75 (Table 1). Regarding acetate formation, the experimental data show an accumulation towards the end of the batch phase after around 12–16 h, which was also addressed in the modeling data.

After finishing the batch phase, the fed-batch phase was initiated with a glucose feeding. In all bioreactor experiments, the feeding growth rates of 0.075, 0.15, 0.2 and 0.25 1/h showed a constant glucose level below the lower detection limit (0.05 g/L) of the enzymatic assay kit, indicating no substrate accumulation. Figure 1 provides an example with feeding growth rates of 0.075 and 0.25 1/h. In the modeled data, glucose remained constant for these feeding rates, including a feeding growth rate of 0.3 1/h. However, in the experimental fed-batch approach using a 0.3 1/h feeding growth rate, a glucose accumulation of up to 13.11 g/L could be observed after 4.75 h of feeding, which declined to 0.08 g/L at the process end, leading to an RMSE of 6.78 (Table 3). For the experimental fed-batch cultivation using a 0.4 1/h feeding growth rate, glucose accumulation was observed immediately after feeding start, reaching about 53 g/L at the process end. The model also showed a glucose accumulation of up to about 23% more compared to the experiment, resulting in an excess of 65 g/L (Figure S1B) and an RMSE of 13.19 (Table 3). Overall, the model simulated all the 12 experimental feeding-phases mentioned before with an average RMSE of 3.59 for glucose.

**Table 3:** Root mean squared error (RMSE) values of the kinetic model for the process parameters biomass X, glucose S, surfactin P and acetate A of the exponential feeding growth rate experiments with feeding growth rates of 0.075, 0.15, 0.2, 0.25, 0.3 and 0.4 1/h

Feeding rate (1/h)	RMSE X	RMSE S	RMSE P	RMSE A
0.075 0.15 0.2 0.25 0.3 0.4	4.66 2.28 1.23 2.14 1.63 2.91	0.56 0.30 0.15 0.53 6.78 13.19	2.41 0.86 2.48 1.35 2.30 0.82	0.08 0.15 0.16 0.26 0.69 2.18
**Mean**	**2.48**	**3.59**	**1.70**	**0.59**

Regarding the biomass formation during the experimental feeding phase, an exponential cell growth could be determined for feeding growth rates of 0.075, 0.15, 0.2, 0.25, and 0.3 1/h, which matched the set exponential rates (Fig. 1 and Table S2). The model simulated these experiments with the same slope as observed in the experiments, resulting in RMSE values between 1.23 and 4.66. For the feeding growth rate of 0.4 1/h, the kinetic model predicted a sigmoidal behavior, which was observed only in one of the experimental duplicates. However, the final biomass value at the end of the process was within a deviation of less than or equal to 5% of the experimental value. Overall, an average biomass-associated RMSE of 2.48 was calculated across all 12 experimental fed-batch experiments.

The growth-associated target product, surfactin, followed the biomass growth after the initiation of the respective feed. The kinetic model accurately reflected this behavior (Fig. 1 and Table S2), resulting in RMSE values ranging from 0.82 to 2.48, with an average RMSE of 1.7 across all model simulations (Table 3). Both the model and experiments reached a maximum surfactin titre at a feeding growth rate of 0.25 1/h. Specifically, the model predicted the highest surfactin amount of 36.47 g/L, while the experiments showed an average maximum of 36.75 g/L.

The biomass productivity remained constant after the feed start for all the tested feeding growth rates, although lower than in the batch phase, as shown exemplarily in Fig. 1 for 0.075 and 0.25 1/h. In the model data for feeding rates of 0.075, 0.15, 0.2 and 0.25 1/h, the by-product acetate, which is part of overflow metabolism, was degraded immediately after the feed start and remained close to 0 g/L throughout the feeding procedure (Fig. 1 and Table S2). These acetate kinetics were also observed in the experimental data for feeding growth rates of 0.075, 0.15 and 0.2, while a slight accumulation of acetate of up to 0.6 g/L could be detected for the feeding growth rate of 0.25 1/h immediately after feed start, which was not simulated by the model (Fig. 1 and Table S2). For the feeding growth rate of 0.3 1/h, the model predicted a moderate degradation of acetate after feed start, which led to a constant acetate concentration of around 0.7 g/L. The experimental data showed a similar level of acetate that accumulated to 2.9 g/L at the process end (Figure S1A). For the feeding growth rate of 0.4 1/h, the model simulated acetate levels up to 6.5 g/L and the experiment showed a maximum of 3.04 g/L (Figure S1B), resulting in a maximum RMSE value of 2.18 for acetate at this feeding rate (Table 3). Overall, an average RMSE for acetate, across all the 12 model simulations, was 0.59 (Table 3).

### Model validation

As described in the Materials and Methods section, a carbon balance was carried out to assess the internal consistency of the model. This analysis was exemplarily performed for a simulation with a feeding growth rate of 0.25 1/h. Based on the simulated end point masses of biomass, surfactin and acetate from Table S2 as well as the total substrate consumption, a carbon recovery of 58.3% was obtained. Specifically, the carbon equivalents were calculated as 110 mol C from glucose, 32.9 mol C in biomass, 31.2 mol C in surfactin and 0 mol C in acetate.

### Sensitivity analysis

To identify the most influential model parameters, a global sensitivity analysis was performed using the Morris method. A feeding growth rate of 0.25 1/h was used as an exemplary condition to evaluate the model structure and its parameter influence. In total, 17 model parameters were varied within a range of ± 10% around their respective reference values. The mean absolute elementary effect ${\mu }^{*}$ was calculated for each parameter and for four objective functions, namely biomass (X), substrate (S), product (P) and acetate (A). The results of the sensitivity analysis are provided in Table S3 in the supplementary material. Among all parameters, the maximum specific growth rate ${\mu }_{max}^{S}$ had the highest influence on model outputs, particularly for biomass, substrate and product. The maximum acetate formation rate b_max_ also showed a considerable impact, especially on biomass, product and acetate. In addition, a strong sensitivity was detected for the substrate-related maintenance parameter m_S_, affecting all model outputs strongly. This clearly highlights the system`s strong dependence on substrate maintenance. Moreover, the yield coefficients significantly influenced system behavior, especially regarding biomass and product formation. In contrast, some parameters, such as acetate-related maintenance coefficient m_A_ or the duration of the lag phase t_Lag_, exhibited only a minor influence on model outputs. This is reflected by their relatively low ${\mu }^{*}$ values under the tested conditions.

### Model-based process design

**Fig. 2:**
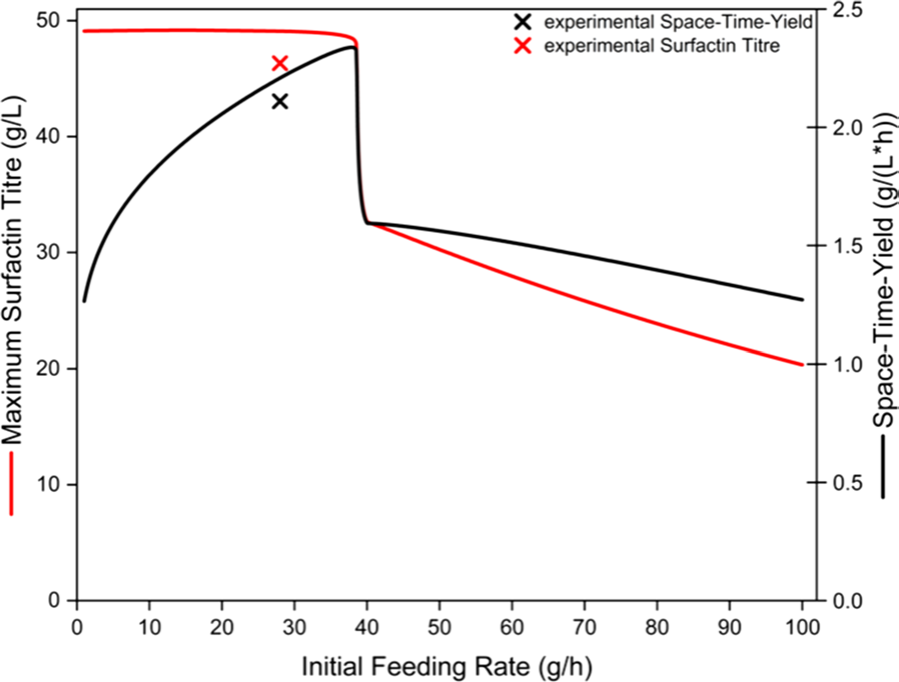
Prediction of the kinetic model in terms of initial feeding rate and the resulting maximum surfactin titre as well as space–time-yield in comparison to the experimental results. Different initial feeding rates for an exponential feeding strategy with a feeding growth rate of 0.2 1/h were utilized by a kinetic model to predict the surfactin titre (red line) and the corresponding space–time yield (black line). The experimentally achieved values are displayed with crosses

After the kinetic model was parameterized with the 12 fed-batch bioreactor experiments, it was subsequently used for the model-based process design. In this process, the batch phase was omitted in order to reduce the overall process time. In literature, the initial feeding rate for the exponential addition of the glucose feed solution after completing the batch phase was calculated for each bioreactor experiment (Klausmann et al. 2021a; Hiller et al. 2024). However, for designing an enhanced process, the kinetic model was used to predict the appropriate initial feeding rate with the aim of achieving the highest possible surfactin titre in combination with a high space–time-yield. Figure 2 shows the progression of the model-predicted maximum surfactin titre and the correlated space–time-yield at the corresponding initial feeding rate in the range of 1 to 100 g/h. Additionally, Table S2 provides the associated process time, the biomass and glucose concentrations reached after the addition of the 10 L of feed solution, as well as the final volume in the bioreactor for each single initial feeding rate.

For the batch phase-free bioreactor process under continuous feeding, a feeding growth rate of 0.2 1/h was selected as it represents a moderate range and ensures that no glucose accumulation or excessive acetate formation is to be expected. The kinetic model predicted in the range of 1 to 38.5 g/h initial feeding rate maximum surfactin titres between 49.17 and 48.01 g/L, with a slight peak of 49.17 g/L surfactin at an initial feeding rate of 14.7 g/h (Fig. 2). The tendency remained almost constant up to an initial feeding rate of 38.5 g/h, and only after exceeding this value did the maximum predicted surfactin titre decrease and reach 32.65 g/L of surfactin at an initial feeding rate of 40 g/h. A reduced final surfactin titre of 20.34 g/L at a initial feed rate of 100 g/h was predicted. In contrast to the trend of the maximum surfactin titre predicted by the kinetic model, the space–time-yield showed a more significant increase in the range of 1 to 38.5 g/h, from 1.26 to 2.32 g/(L*h) (Fig. 2). The model predicted a peak space–time-yield of 2.34 g/(L*h) at an initial feed rate of 38 g/h. Up to 40 g/h, a decline of the space–time-yield to 1.59 g/(L*h) was predicted. From this initial feeding rate, a continuous decrease of the space–time-yield was observed reaching a minimum of 1.27 g/(L*h) at an initial rate of 100 g/h. Thus, the kinetic model predicted very similar trends for both process parameters, surfactin titre and space–time-yield (Fig. 2).

To test the predictive power of the kinetic model, an initial feed rate of 28 g/h was selected. This rate was predicted to be approximately 25% below the maximum space–time-yield and combined with the advantages of a moderate model-predicted process time of around 22 h and an expected surfactin titre of about 49 g/L (Fig. 2). At the same time, the model predicted an absence of glucose accumulation towards the end of the experiment. Thus, an excessive formation of by-products, such as acetate, could be avoided (Table S2).

**Fig. 3:**
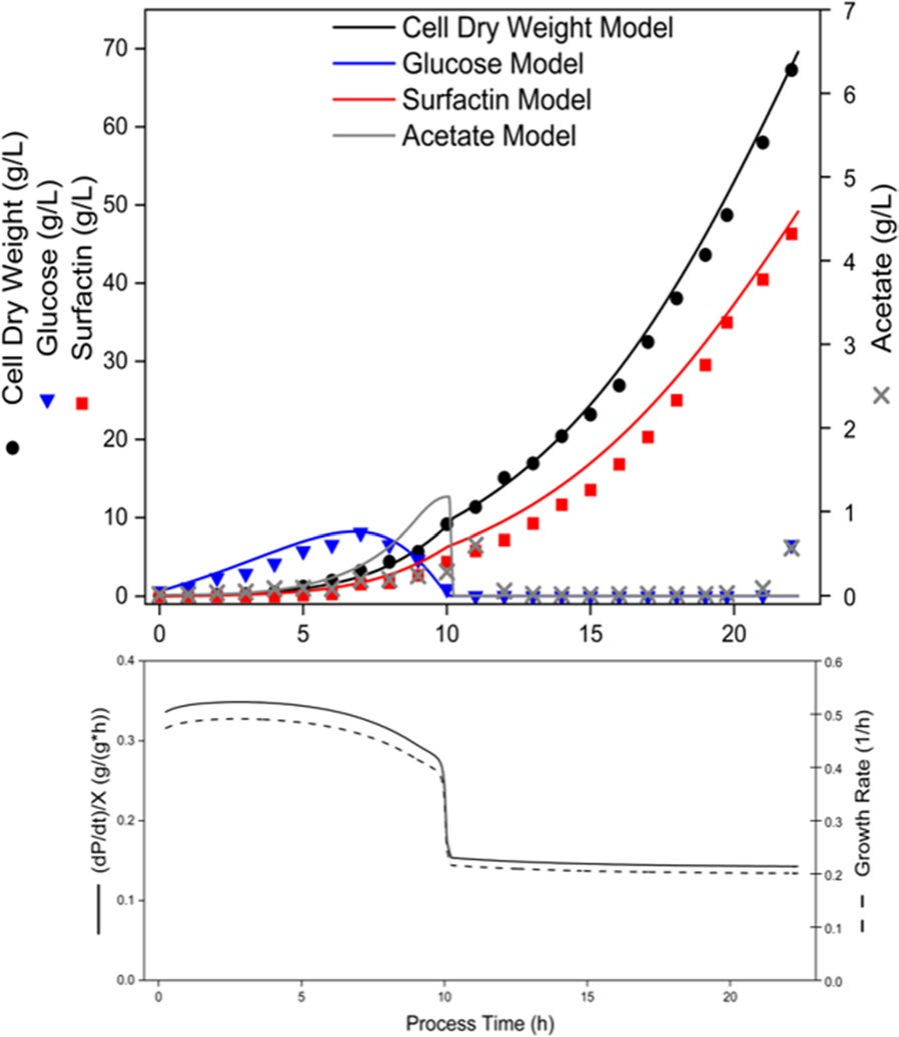
Time course of a model-based process design cultivation of *B. subtilis* BMV9 and modeling of the data. Measured parameters, were cell dry weight (black circles), glucose (blue inverted triangle), surfactin (red squares) and acetate concentration (grey crosses). The lines through the data points of cell dry weight, glucose, surfactin and acetate represent the model prediction. Additionally, the specific growth rate of biomass (black line) on the substrate glucose and the biomass productivity (black dashed line) are continuously represented. These values were calculated using the model

**Fig. 4:**
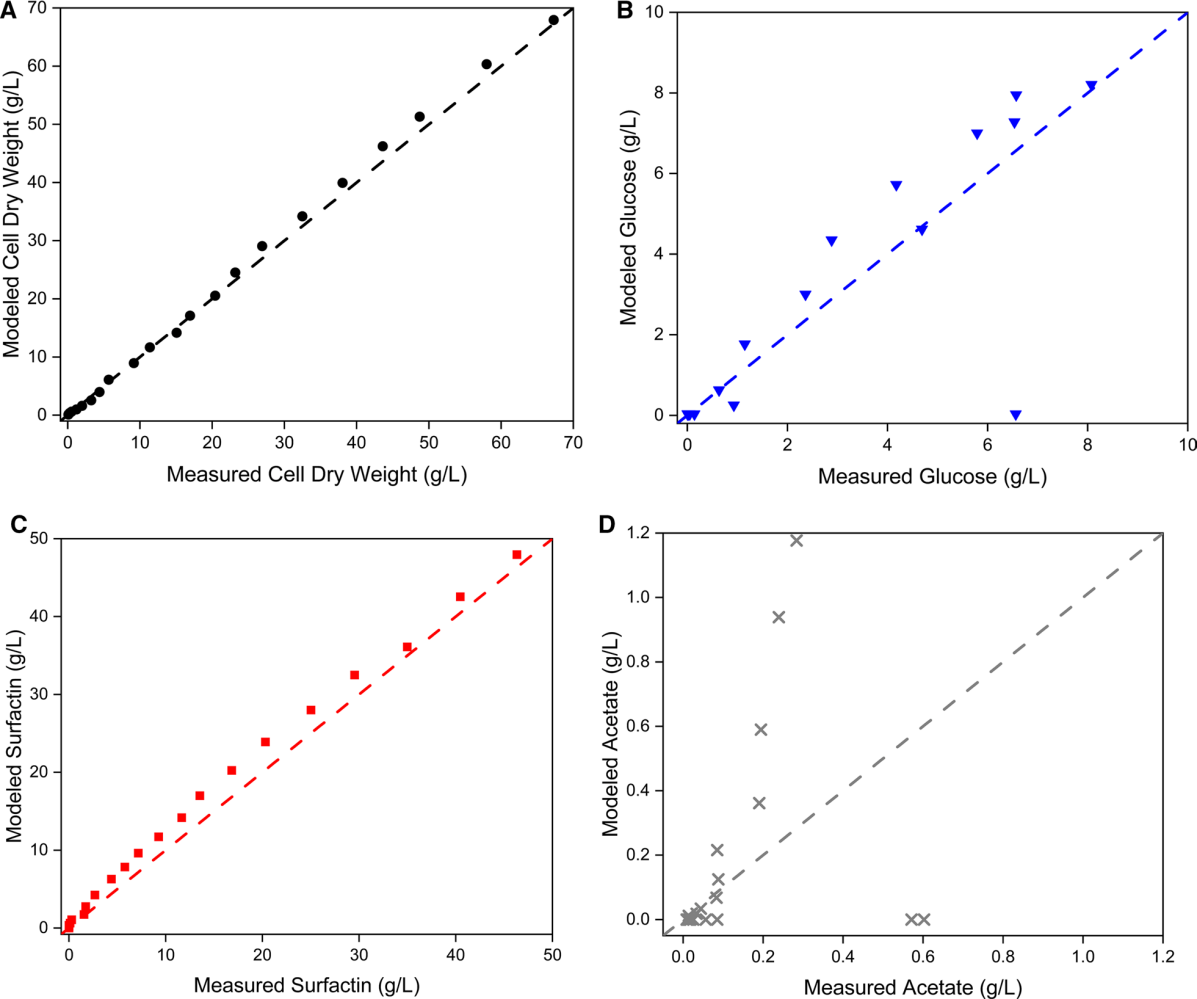
Comparison of the experimentally determined and model-generated values of cell dry weight (**A**) glucose (**B**) surfactin (**C**) and acetate (**D**) for the model-based process design. Parameters, were cell dry weight (black circles), glucose (blue inverted triangle), surfactin (red squares) and acetate concentration (grey crosses). The dashed lines represent the bisecting lines, which indicate the ideal concordance between experimental and modeled values

The temporal experimental profiles of cell dry weight, glucose, surfactin and acetate concentrations, as well as the modeled growth rate and the cellular productivity of the model-based process design are shown in Fig. 3. In more detail, an initial feeding rate of 28 g/h was applied both in the simulated data (solid lines) and in the experiment (data points), after a defined amount of cells from a preculture were added to the pre-supplied salt medium (without glucose) in the bioreactor. Figure 4 shows the comparison between experimentally determined and model-generated values for cell dry weight, glucose, surfactin and acetate by plotting them against each other, including the bisecting line which indicates the ideal concordance between model and experiment. Using the RMSE, the statistical accuracy of the kinetic model-based prediction for this experiment without a batch phase is provided for each process parameter in Table 4. Moreover, Table 5 summarizes the specific productivities and yields, along with the experimentally achieved space–time-yield and the maximum surfactin titre reached.

**Table 4:** Root mean squared error values of the kinetic model for the process parameters biomass X, glucose S, surfactin P and acetate A of the model-based process design with a feeding growth rate of 0.2 1/h

RMSE X	RMSE S	RMSE P	RMSE A
1.19	1.51	2.00	0.31

**Table 5:** Evaluation of the maximum product titre, the time–space-yield, the specific productivities and the production yields for the surfactin production with *B. subtilis* BMV9 in a fed process. All values were calculated for the model-based bioreactor experiment with a feeding growth rate of 0.2 1/h

Maximum titre (g/L)	Y_P/S_ (g/g)	Y_P/X_ (g/g)	Y_X/S_ (g/g)	q_P/S_ (g/(g*h))	q_P/X_ (g/(g*h))	P_V_ (g/(L*h))
46.33	0.20	0.69	0.29	0.01	0.03	2.11

The initial conditions for the model-based simulation were given by the amount of biomass, glucose, surfactin and acetate provided by the preculture. Thus, minimal difference between the experimental values and the simulated data was observed at the beginning. A comparison of the total process time between simulation and experiment showed a difference of 0.23 h, which means a deviation of only 1% for a process time of 22 h.

The analysis of the glucose profile revealed a very similar trend in both the model and the experiment. Due to the underlying adaptation of the cells to the bioreactor conditions, a short lag phase was represented in the model, which led to an accumulation of the substrate concentration immediately after the start of the process. This phenomenon was also observed in the experiment. Subsequently, the simulated glucose concentration reached a peak of 8.23 g/L after 6.78 h (Table S2 and Fig. 3). In comparison, the experimental kinetics resulted in a maximum glucose concentration of 8.07 g/L after 7 h (Fig. 3). In total, a deviation of only 0.16 g/L could be found between the experimental and the simulated data, with a time difference of 0.22 h. A subsequent decline resulted in final glucose concentrations of below 1 g/L after 10 h of cultivation in both the experiment and the model (Fig. 3). Afterwards, the glucose concentration remained close to the lower detection limit in both the experimental and simulated data. Only at the end point of 22 h, a slight increase to 6.56 g/L was observed in the experimental data, which was not reflected in the model. The very similar trends between the experiment and the model were also reflected by an RMSE value of 1.51 (Table 4) and data points close to the bisecting line (Fig. 4B). The greatest deviation was also observed for the final glucose value after 22 h of process time, where an increase to 6.56 g/L was measured in the experiment. However, this increase was not represented in the model, causing this data point to deviate significantly from the bisecting line (Fig. 4B).

Regarding the biomass (CDW) kinetics, an overlapping outcomes could be observed in both the experiment and the simulated data. After an initial lag phase, cell growth started at the same time of 0.25 h in both the experiment and the simulation. After the initially accumulating glucose could be consumed after around 10 h of process time, a decrease in the biomass growth rate was observed in both the experimental data and the model-based simulation. Accordingly, an adaptation of the modeled growth rate was available in relation to the feeding growth rate of 0.2 1/h (Fig. 3). The final biomass concentration in the bioreactor experiment reached 67.29 g/L after 22 h, while the model predicted a concentration of 67.93 g/L at the same time. This similarity in trends was reflected in the RMSE value of 1.19 (Table 4). This trend is also depicted in Fig. 4A, as nearly all data points are located very close to the bisecting line.

For the target product surfactin, the production kinetics followed the biomass growth in both the simulated data and the experiment. This was also demonstrated in the trend of the productivity, (dP/dt)/X, which closely mirrored the cell growth rate. After the lag phase, a simulataneous increase in biomass and surfactin was detectable, resulting in a productivity of approximately 0.35 g/(g*h) as long as an excess of glucose was available. After consumption of the initially accumulated glucose at around 10 h of cultivation, a decrease of the productivity to about 0.17 g/(g*h) could be shown. Finally, the model predicted a maximum surfactin titre of 49.16 g/L after depletion of the glucose feed solution of 10 L. In comparison, the experiment allowed a similar outcome of 46.33 g/L, showing a deviation of 5.7% from the simulation (Figs. 2, 3 and Table 5). In this context, comparable values for the space–time-yield could be calculated, with a model prediction of 2.21 g/(L*h) and an experimental outcome of 2.11 g/(L*h), which led to a deviation of 4.5% (Figs. 2, 3 and Table 5). The similarity was also reflected in an RMSE value of 2.00 (Table 4). This is also confirmed in Fig. 4C, as the data points are very close to the bisecting line and exhibit a linear behavior.

In examining the progress of the by-product acetate, which was considered a representative of overflow metabolism in the model, a similar pattern was observed in both the simulation and the experiment. In the experiment, the acetate concentration remained below 0.1 g/L during the first 6 h of cultivation, followed by an increase of up to 0.6 g/L after 11 h, and subsequent decrease to almost 0 g/L after the excess glucose was consumed. For the rest of the process, the acetate concentration remained at 0 g/L. This leads to a large accumulation of data points at 0 g/L in Fig. 4D. However, after the slight glucose increase after 22 h of cultivation, the acetate concentration also showed slightly increase of up to 0.57 g/L at the end of the experiment (Fig. 3). In comparison to the experimental acetat kinetic, a similar trend was predicted in the model. Nevertheless, noticeable differences could be found in the maximum acetate value and the time course of acetate accumulation and depletion, respectively (Fig. 4D). In more detail, the simulated data showed an acetate concentration of 1.18 g/L after 10 h, which was 1 h earlier than the experimental data and twice as high as the experimental level of 0.6 g/L. In addition, no final acetate accumulation was predicted in the model at the end of the process. Despite these differences, the overall acetate trend was well captured in the model, as reflected in an RMSE value of 0.31 (Table 4).

In addition to the parameters of space–time-yield and maximum surfactin titre mentioned before, the production yields and specific productivities were also calculated for the model-based experiment. The results are presented in Table 5.

### Limits of the model prediction

To test the limitations of the predictive power of the kinetic model, the model-based process design (Fig. 3) was continued with 5 L of glucose feed solution, allowing a total feed of 15 L. This is thus associated with a 50% increase in the amount of glucose, reaching the maximum reactor fill level with the additional feeding volume. In consequence, the process time was extended by 2 h, resulting in a total experimental duration of 24 h. The experimentally collected results as well as the model-generated data for CDW, glucose, surfactin and acetate during these additional 50% glucose amount are presented in Table S4. Regarding the biomass, a visually noticeable morphological change in the cells was noticed in the experiment but a deviation of less than 1 g/L was observed between the model prediction and the experiment after 23 h of cultivation. However, due to an overestimation of the model prediction compared to the experimental data, an increase in deviation of up to 2.31 g/L was shown after 24 h of cultivation. In this context, an accumulation of glucose was detected after 22 h of cultivation, leading to a final concentration of 46.29 g/L at the end of the process. In contrast, the model continued to show glucose concentrations close to 0 g/L, even with the extension to 15 L of feed solution. Regarding surfactin, the model predicted an increase of up to 59.07 g/L after 24 h. In the experiment, however, a total surfactin concentration of 0.40 g/L was measured. In fact, there was a product degradation that is not represented in the model, which leads to an erroneous assessment by the model. In the case of acetate, the model-based prediction showed a constant level up around 0 g/L with an increase in feed volume. In the experiment, the acetate concentration increased slightly, yielding a measurement of 0.61 g/L after 23 h, and 0.33 g/L after 24 h. Overall, the model did not capture the glucose accumulation and the acetate trend satisfactorily when the feed volume exceeded 10 L.

To demonstrate the reproducibility of the model-based process design, the bioreactor experiment shown in Fig. 3 was adapted in terms of the preculture used for inoculation (Figure S2). Therefore, a preculture was used, which led to different initial conditions for the bioreactor experiment and the model, namely a half as high initial CDW, an approximately twice as high residual glucose concentration and an approximately three times as high acetate concentration. These inoculation conditions led to a 25% extended lag phase in the model compared to the bioprocess described in Fig. 3 in order to best fit the experimental data. The resulting glucose accumulation with a RMSE of 2.24, cell growth with a RMSE of 1.91 and surfactin production with a RMSE of 0.54 were relatively well described by the model with the extended lag phase (Table S2 and Figure S2). However, acetate production was measured at 0.39 g/L after 13 h of cultivation, while the model predicted an acetate level of 2.65 g/L at the same time (Table S2 and Figure S2). Additionally, the trend of acetate concentration over the entire process time was not captured by the model. At the beginning of the process, acetate accumulated slowly for about 8 h, with identical trends observed in both the model and the experiment. However, as the process progressed, the model predicted a sharp increase in acetate concentration to approximately 3.5 g/L after 14 h, while only 0.39 g/L was measured in the experiment at the same time. After this sharp increase, the model shows a nearly linear decline and after around 22 h, the acetate concentration abruptly drops to 0 g/L. In contrast, in the experiment, approximately 0 g/L acetate was reached after 15 h, and this concentration remained unchanged until 20 h. After that, acetate increased to 1.39 g/L at 22 h. This is reflected in a RMSE of acetate of 1.92. This situation demonstrates the strong dependence of the predictive power of the model on the pre-culture conditions in the experiment and thus another limitation in the description and prediction of the overflow metabolism.

## Discussion

In previous studies, the focus was on the general development of a high-cell-density fed-batch process with an exponential feeding strategy for the production of surfactin using *B. subtilis* in a bioreactor (Klausmann et al. 2021a). On this basis, the effect of the feeding growth rate in a range of 0.075 to 0.4 1/h on the production efficiency of surfactin was investigated, resulting in a product titre of 36.75 g/L (Hiller et al. 2024). However, these studies lacked a basic understanding of the underlying kinetics. To address this issue and gain deeper insights into biomass growth, substrate consumption, surfactin production and by-product formation through overflow metabolism, a kinetic model was developed in this study. The model was parameterized using fed-batch bioreactor experiments and ultimately used to derive an enhanced process design. In the parametrization process, it is particularly important that all parameters are fitted simultaneously in order to capture the complex interactions between biomass growth, substrate consumption, product formation and overflow metabolism.

The modeling of the 12 fed-batch bioreactor experiments from Hiller et al. (2024) provided a deeper understanding of the underlying kinetics related to biomass formation, substrate consumption, product formation and overflow metabolism. In this way, a kinetic model could be established, allowing the calculation of productivities, yields and biomass growth rate as biological performance indicators at any point in the process time. The kinetic model thus makes important key performance indicators accessible at any point in the process and can relate them to each other as required. For example, Fig. 1 shows that the productivity of the biomass is strongly dependent on the glucose concentration present in the bioreactor. This is particularly noticeable in the batch phase, in which higher glucose concentrations lead to increased productivity. During the feeding phase, productivity remains constant, but at a lower level, depending on the growth rate. By making the biological performance indicators accessible over the entire process period, such maxima can be recognised using the kinetic model. This allows completely new process modes to be developed in which the feed is controlled online by the kinetic model and kept at a fixed glucose concentration level. Knowledge of the kinetics, which contribute to a better understanding of the process, is essential for this. To validate the kinetic model, the carbon recovery was calculated. A value of 58.3% indicates that a substantial portion of the substrate-derived carbon was captured in biomass and product. The unrecovered fraction is most likely attributable to carbon dioxide formation through cellular respiration and the synthesis of minor metabolic by-products, both were not included in the kinetic model. To further evaluate the reliability and robustness of the developed kinetic model, a global sensitivity analysis was conducted using the Morris method. The analysis revealed that only a subset of parameters had a strong influence on model outputs, particularly the maximum specific growth rate on substrate, the maximum acetate formation rate and the substrate related maintenance coefficient. These parameters showed consistently high absolute elementary effect values across all model targets, namely biomass, substrate, product and acetate. This highlights their central role in determining process dynamics. In contrast, other parameters like the maintenance coefficient on acetate or the duration of the lag phase showed only marginal effects under the tested conditions, indicating model robustness with respect to these variables. These results underscore the importance of accurate estimation and experimental validation of the most sensitive parameters in order to ensure reliable model predictions and meaningful interpretation of biological behavior. Moreover, the findings provide valuable guidance for future optimization and model-based control strategies, because highly influential parameters represent ideal targets for focused experimental design.

Using the model-based enhanced process design, a surfactin titre of 46.33 g/L was achieved. Therefore, a feeding growth rate of 0.2 1/h was selected, which is close to the maximum growth rate of *B. subtilis* BMV9 under the given conditions according to Hiller et al. (2024), without provoking overflow metabolism due to glucose accumulation. As a result, the new process design is comparable to the production yields and specific productivities reported by Hiller et al. (2024) for a feeding growth rate of 0.2 1/h. The production yield Y_P/X_ in the model-based process was 6.2% higher, Y_P/S_ showed minimal deviation, and Y_X/S_ was 3.6% higher in comparison to the feeding phase of the traditional fed-batch experiment. When comparing the specific productivity of the biomass, meaning the q_P/X_ value, was the same value as reported for the fed-batch process in Hiller et al. (2024). In the case of q_P/S_ an 20% higher value was achieved in the enhanced process design when comparing to the original fed-batch process. The maximum surfactin titre was increased by 35% using the model-based approach, while the overall process time was reduced by almost 2 h, although the feeding volume was increased by 67% (Hiller et al. 2024). Compared to other studies with bioreactor production processes using *B. subtilis*, the maximum surfactin titre was increased by 28% (Amin 2014) and 75% (Klausmann et al. 2021a). Comparing the space–time-yield P_V_, the percentage increase relative to the fed-batch process with a feeding growth rate of 0.2 1/h was high. Hiller et al. (2024) achieved a P_V_ value of 1.45 g/(L*h), while the model-based enhanced process exceeded this value by 61%. The comparison of the model-based enhanced process design with process parameters from other fed-batch bioreactor processes in literature shows clear advantages of omitting the batch phase. First, a high initial substrate concentration during inoculation leads to an extension of the lag phase, resulting in an unproductive process phase in terms of cell growth and therefore product formation. This was only shown for shake flask cultivations before by Willenbacher et al. (2015), by using different initial glucose amounts. In contrast, Fig. 3 demonstrates an favourable alternative in this regard. The biomass growth rate reached a maximum of nearly 0.5 1/h after 0.25 h, while the batch phase in Hiller et al. (2024) showed hardly any increase in CDW during the first 4 h after inoculation. Presumably due to adaptation effects or substrate inhibition. Similarly, the *B. subtilis* strain JABs32, from which BMV9 is derived, also showed minimal growth and surfactin production during the first 6 h of cultivation (Klausmann et al. 2021a). Both processes provided an initial glucose concentration of 25 g/L, almost 50 times more than the amount of glucose introduced into the medium of the new model-based process by the preculture as an artifact.

In various studies, the growth kinetics of biomass has been modeled using Monod kinetics without any inhibition terms. Examples include the growth of *Aspergillus niger* for sodium gluconate production and *B. subtilis* for modeling lipopeptide production (Dong et al. 2015; Guez et al. 2007). However, Alvarado et al. (2022) demonstrated that omitting an inhibition term in biomass growth kinetics for *B. subtilis* biosurfactant production can result in up to five times greater error. Accordingly, in this study, the Monod kinetics was extended with the general inhibition term described by Stepanova and Romanovskii (Yano and Koga 1973), which was applied to the overflow metabolite acetate. This modification led to a mean RMSE value of 2.48 (Table 3) across all 12 experiments, resulting in highly accurate biomass simulation for different feeding growth rates. This value was even surpassed with an RMSE of 1.19 (Table 4) in the model-based enhanced process, highlighting the predictive capability of the model regarding biomass growth. The importance of implementing biomass growth inhibition through overflow metabolism is further emphasized by the attempt to reduce model complexity via the exclusion of acetate-related dynamics. This model reduction, which involved omitting acetate formation, acetate-associated substrate consumption and the inhibitory effect on biomass growth, resulted in biologically implausible behavior during parametrization. These findings demonstrate that neglecting key by-product pathways in the interest of simplification can significantly impair model performance and predictive power, underlining the necessity of including overflow metabolism for an accurate description of the system. This outcome highlights the importance of accounting for inhibitory by-products of overflow metabolism when modeling bioprocesses. The production of biosurfactants by *B. subtilis* has often been described in the literature using the Luedeking-Piret equation (Ludeking and Piret 1959). In the case of surfactin, the production is clearly linked to cell growth. In this context, the experimental data from Hiller et al. (2024) and this study confirm this observation and clearly demonstrate a relation between surfactin production and cell growth. This was reflected in the kinetic model, when the non-growth-associated term of the Luedeking-Piret equation was set to zero. This approach resulted in RMSE values of 1.70 for the 12 fed-batch experiments and 2.00 for the model-based experiment, highlighting that the model effectively captures growth-associated surfactin production.

Despite the simulation of biomass and surfactin production in both the 12 fed-batch bioreactor experiments and the model-based enhanced process in this study, the model showed considerable limitations when overflow metabolism was stimulated in the presence of relatively high glucose concentrations. This was particularly evident at feeding growth rates of 0.3 and 0.4 1/h, where increased acetate formation was observed (Hiller et al. 2024). This issue became noticeable in the model-based experiment with an increased feed volume from 10 to 15 L (Table S4). This leads to the conclusion that the kinetic model developed in this study clearly shows, that there is a need of analyzing all overflow metabolites of *B. subtilis*, although Presecan-Siedel et al. (1999) demonstrated that acetate is one of the most abundantly produced by-product of *B. subtilis* carbon metabolism. However, for moderate feeding growth rates of up to 0.25 1/h, at which efficient surfactin production could be measured, the simplification of the overflow metabolism to acetate and the associated inhibition caused by the model seems to be sufficient (Fig. 1). This allows the simulation of the production process using two threshold values for the glucose concentration and a linear increase in the acetate formation rate up to a maximum limit. It also supports the establishment of a novel batch-free process with a significantly higher surfactin titre and a lower overall process time. To improve the limitations of the kinetic model, it is essential to more accurately describe the overflow metabolism. This would explain phenomena such as an accumulation of glucose at a feeding growth rate of 0.3 1/h and the reduction of biomass growth observed when the feed volume is expanded (Figure S1, S2). For an appropriate bioprocess-dependent clarification of *B. subtilis* overflow metabolism, extensive measurements need to be performed for potentially inhibitory (overflow) metabolites. A deeper understanding of the by-product formation, degradation and potential interaction is crucial for refining the model and better predicting process behavior under substrate accumulating conditions. Modeling these conditions will provide a deeper understanding of the surfactin production process.

In addition to acetate, several other overflow metabolites produced by *B. subtilis* were reported, particularly volatile compounds. These include substances like acetoin or butanediol (Kabisch et al. 2013; Dettwiler et al. 1993). Speck and Freese (1973) showed that acetate induces the enzymes responsible for acetoin and butanediol production, significantly accelerating acetoin formation in *B. subtilis*. This could explain the drop in glucose after an initial increase at a feeding growth rate of 0.3 1/h, as excessive acetoin production might be triggered at that point and therefore a high amount of the substrate was consumed (Figure S1). In addition to the previously mentioned overflow metabolites, propionate is also frequently discussed, which has a similar, but weaker, accelerating effect on acetoin and butanediol production (Speck and Freese 1973). López et al. (1975) demonstrated that acetoin can be further oxidized through dissimilation into acetate. This creates a feedback loop in which acetate production is accelerated and may explain the steep increases in acetate concentration observed at higher feeding growth rates. This phenomenon suggests that the interplay between acetoin and acetate formation contributes significantly to the overflow metabolism in *B. subtilis*, particularly under conditions of high glucose availability. The inhibitory effect of acetate on the *B. subtilis* cells in the pre-culture could also explain an extended lag phase and higher glucose accumulation in the bioreactor, which would additionally trigger the overflow metabolism, in the initial cultivation phase of the model-based enhanced process with extended lag phase (Figure S2). Accordingly, an increase in the acetate levels, the cell growth would be reduced or inhibited, leading to a reduced metabolism and, consequently, an accumulation of the continuously fed glucose. This accumulation would then intensify the overflow metabolism, resulting in further by-product formation, which complicates the overall process dynamics. Another by-product of the *B. subtilis* overflow metabolism, which can be formed from pyruvate, the precursor to acetoin, is lactate (Dettwiler et al. 1993). Additional overflow metabolites produced by *B. subtilis* include ethanol, succinate, formate, isobutyrate, and isovalerate (Suárez et al. 2024). These compounds are mainly formed under conditions of substrate excess, which can lead to inefficient energy and substrate utilization by the cells.

Simple kinetic models can not fully capture the complex interactions and nuances of bioprocesses (Mears et al. 2017). The difficulty lies in describing the essential influencing variables as simply as necessary. Parameters are often pre-determined or fitted to decrease the complexity of the system, using empirical data to approximate some of these values. This approach allowed the model to focus on the most critical aspects of the process, avoiding the need to account every detailed mechanism (Andrews 1993). The result was a model that was less computationally intensive and more practical for tasks like model-based process optimization and process design. In this way, a more detailed understanding was not required for every component in the bioprocess. Alternative modeling strategies for *B. subtilis* processes, such as genome-scale metabolic models, structured models or hybrid data-driven approaches, have been explored in literature (Blázquez et al. 2023; Massaiu et al. 2019; Winz et al. 2023). However, these type of models require large scale omics data, detailed intracellular information or advanced computational tools. The kinetic model presented in this study instead aims to capture the essential process dynamics with minimal complexity, focusing on parameters that are both experimentally accessible and relevant for fed-batch surfactin production. This makes it a robust tool for process design.

In summary, it is crucial to further improve the kinetic model presented in this study with regard to overflow metabolism. Special attention should be given to the dual feedback loop between acetate and acetoin. Additionally, the variety of metabolites identified should be considered to better describe processes under glucose excess and to develop a deeper understanding of the underlying kinetics. Nevertheless the usage of the general inhibition term described by Stepanova and Romanovskii seems to be a good choice for *B. subtilis* (Yano and Koga 1973). Despite that, the model described here is able to satisfactorily describe the development of the *B. subtilis.* BMV9 biomass quantity, the substrate and product quantities under the selected production conditions and even to interpolate within the given boundaries.

## Conclusion

This study demonstrates the successful development of a kinetic model for surfactin production in fed-batch bioreactor processes which significantly contributes to a higher process understanding according to *Bacillus subtilis* biomass formation, product synthesis as well as substrate utilization kinetics. The model provided critical insights into biological key performance indicators such as production yields, specific productivities and growth rates as well as process kinetics with consideration of the overflow metabolism, enabling the model-based design of an efficient bioreactor process that eliminates the need for a batch phase. In this context, the predictive capability of the kinetic model was demonstrated. However, the study also highlighted challenges related to overflow metabolism, which can lead to the accumulation of by-products such as acetate. Nevertheless, the insights obtained from the *B. subtilis* BMV9 strain can serve as a foundation for developing bioproduct formation processes with other sporulation-deficient *Bacillus* strains.

## Supplementary Information

Supplementary Material 1.

Supplementary Material 2.

Supplementary Material 3.

Supplementary Material 4.

Supplementary Material 5.

Supplementary Material 6.

## Data Availability

The datasets generated for this study are provided in this study and are saved in the Institute of Food Science and Biotechnology, Department of Bioprocess Engineering (150 k), University of Hohenheim, Fruwirthstraße 12, Stuttgart 70,599, Germany. The MatLab codes (files: FedBatchModel_EH.m, TerminationFunction.m, runSimulation.m, RunSimulation_EH.m) of the kinetic model are provided in the supplementary material of the manuscript. In case of requirement, please contact the corresponding author for any detailed question.
