# Supplementary material for: Model-based process design for surfactin production with Bacillus subtilis
Source: AMB Express. 2025 Nov 21;15:179. doi: 10.1186/s13568-025-01978-3 (PMC12712264; doi:10.1186/s13568-025-01978-3)
Supplement: Supplementary file 4 — Supplementary Material 4. [file 13568_2025_1978_MOESM4_ESM.docx]

**Model-based process design for surfactin production with *Bacillus subtilis***

Eric Hiller^1*^ (ORCID ID: 0009-0000-3815-920X), Manuel Off^1^ (ORCID ID: 0009-0009-3585-6822), Holger Dittmann^1^ (ORCID ID: 0009-0009-3383-0610), Elvio Henrique Benatto Perino^1^ (ORCID ID: 0000-0003-0372-260X), Lars Lilge^1*^ (ORCID ID: 0000-0002-7693-477X), Rudolf Hausmann^1^ (ORCID ID: 0000-0002-2327-7120)

^1^ Department of Bioprocess Engineering, Institute of Food Science and Biotechnology, University of Hohenheim, Stuttgart, Germany.

*Corresponding author: [eric.hiller@uni-hohenheim.de](mailto:eric.hiller@uni-hohenheim.de)

**Table S1** Overview of all model parameters and variables, along with their units and definitions

| **Model variable** | **Unit** | **Definition** |
| --- | --- | --- |
| X  X_0_  S  S_0_  P  P_0_  A  A_0_  V  V_0_  v  t  F  F_0_  $\mu_{S}$  $\mu_{max}^{S}$  $\mu_{A}$  $\mu_{max}^{A}$  $K_{S}$  $K_{A}$  $Y_{X/S}$  $Y_{X/S}^{true}$  $Y_{P/X}$  $Y_{P/S}$  $Y_{A/S}$  $Y_{X/S,Batch}$  $Y_{X/A}$  $m_{S}$  $m_{A}$  $t_{Lag}$  $\mu_{F}$  $c_{S, crit}$  $c_{S, Feed}$  $\rho_{Feed}$  X_FS_  t_FS_  $c$  b  $b_{max}$  $c_{S,crit,A1}$  $c_{S,crit,A2}$  $K_{I}$ | g  g  g  g  g  g  g  g  L  L  L  h  g/h  g/h  1/h  1/h  1/h  1/h  g/L  g/L  g/g  g/g  g/g  g/g  g/g  g/g  g/g  g/(g*h)  g/(g*h)  h  1/h  g/L  g/L  g/L  g  h  -  1/h  1/h  g/L  g/L  g/L | Biomass amount  Initial biomass  Substrate amount  Initial substrate  Product amount  Initial product  Acetate amount  Initial acetate  Filling volume of the bioreactor  Initial filling volume of the bioreactor  Feed volume  Time  Feed rate  Initial feed rate  Specific growth rate of biomass X on substrate S  Maximum specific growth rate of biomass X on substrate S  Specific growth rate of biomass X on acetate A  Maximum specific growth rate of biomass X on acetate A  Half-saturation constant substrate S  Half-saturation constant acetate A  Conversion yield of substrate S to biomass X  Theoretical maximum conversion yield of substrate S to biomass X  Production yield of product P by biomass X  Conversion yield of substrate S to product P  Conversion yield of substrate S to acetate A  Conversion yield of substrate S to biomass X in the batch phase  Conversion yield of acetate A to biomass X  Maintenance substrate S  Maintenance acetate A  Duration of lag phase  Feeding growth rate  Substrate concentration for feed start  Substrate concentration in the feed  Density of the feed  Biomass amount at feed start  Time at feed start  Volume correction factor  Acetate formation rate  Maximum acetate formation rate  Critical substrate concentration for the start of acetate formation  Critical substrate concentration for maximum acetate formation  Inhibition constant |


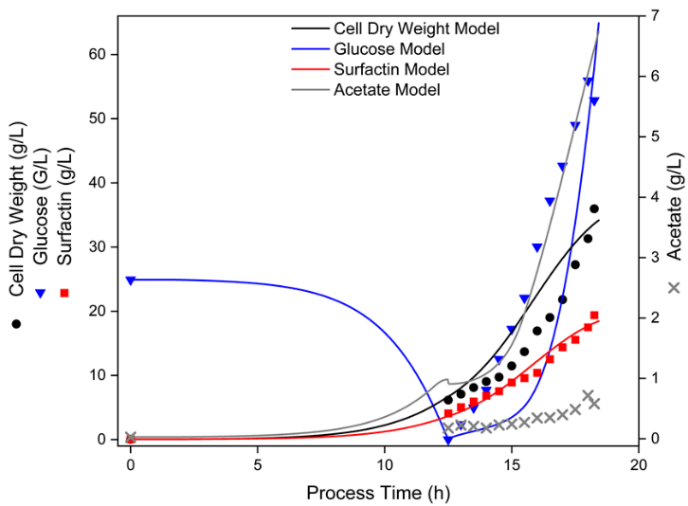

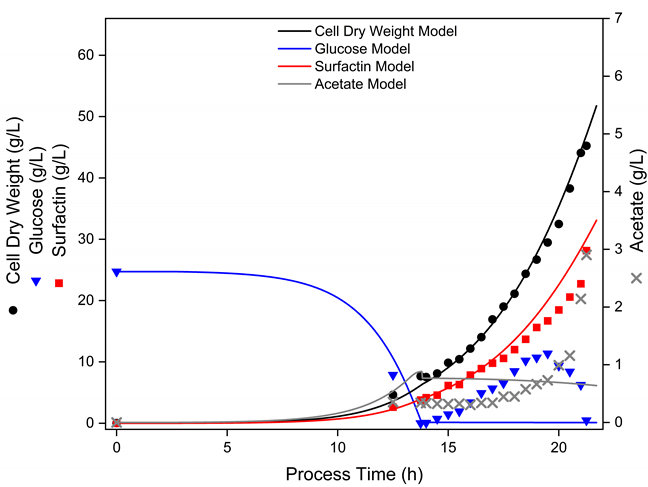


**A**

**B**

**Fig. S1 Time course of bioreactor cultivations using B. subtilis BMV9 with different exponential feeding growth rates and modeling of the data.** After glucose depletion, the batch phase was stopped and the exponential feeding process with a feeding growth rate of 0.3 1/h (A) and 0.4 1/h (B) was started. The fed-batch was finished when the 6 liters of glucose feed solution was depleted. Measured parameters were cell dry weight (black circles), glucose (blue inverted triangle), surfactin (red squares) and acetate concentration (grey crosses). The lines represent the model fit

**Table S3** Results of the global sensitivity analysis using the Morris method. A total of 17 model parameters were varied within a range of ± 10 % around their respective reference values. The mean absolute elementary effect $\mu^{*}$ is reported as the sensitivity measure for biomass (X), substrate (S), product (P) and acetate (A). The analysis was conducted for a feeding growth rate of 0.25 1/h

| **Parameter** | **µ^*^ X** | **µ^*^ S** | **µ^*^ P** | **µ^*^ A** |
| --- | --- | --- | --- | --- |
| $\mu_{max}^{S}$  $\mu_{max}^{A}$  $K_{S}$  $K_{A}$  $Y_{X/S}^{true}$  $Y_{P/X}$  $Y_{P/S}$  $Y_{A/S}$  $Y_{X/A}$  $m_{S}$  $m_{A}$  $t_{Lag}$  $c_{S, crit}$  $b_{max}$  $c_{S,crit,A1}$  $c_{S,crit,A2}$  $K_{I}$ | 221000  384  11.9  0.1021  10.24  5.057  9.082  0.483  0.183  4907  0.00  0.001099  0.988  44200  5.051  0.00544  0.253 | 41600  3.48  0.922  0.000143  5.98  1.67  3.28  0.155  0.000385  7901  0.00  0.000268  0.00983  174  0.0276  0.0005035  0.0697 | 79600  46.003  3.604  0.0331  6.86  11.9  5.40  0.340  0.0139  3690  0.00  0.000662  0.237  42900  0.179  0.00112  0.219 | 14060  1240  8.077  0.0590  0.285  0.4028  0.500  0.000000215  0.1306  2036  0.00  0.00000138  2.61  2280  5.93  0.0214  0.00783 |


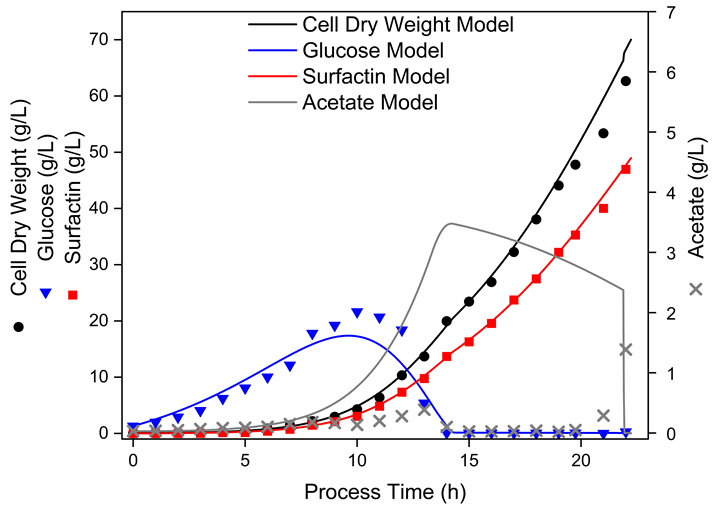


**Fig. S2 Time course of a model-based designed bioreactor cultivation using B. subtilis BMV9 and modeling of the data.** The exponential feeding of the glucose feed started directly after inoculation of the bioreactor, with an initial feeding rate of 28 g/h and a feeding growth rate of 0.2 1/h. The process was stopped when the 10 liters of feed solution was depleted. Measured parameters were cell dry weight (black circles), glucose (blue inverted triangle), surfactin (red squares) and acetate concentration (grey crosses). The lines represent the model fit

**Table S4** Comparison of the measured CDW, glucose, surfactin and acetate with the prediction of the kinetic model after expansion of the glucose feeding volume from 10 to 15 liters. All measurement points are shown after feeding 10 liters in 22 hours exponential feeding

| **Process time (h)** | **CDW (g/L)** | **Model CDW (g/L)** | **Glucose (g/L)** | **Model Glucose (g/L)** | **Surfactin (g/L)** | **Model Surfactin (g/L)** | **Acetate (g/L)** | **Model Acetate (g/L)** |
| --- | --- | --- | --- | --- | --- | --- | --- | --- |
| 23  24 | 76.57  81.21 | 75.71  83.52 | 23.10  46.29 | 0.03  0.03 | 0.51  0.40 | 53.50  59.07 | 0.61  0.33 | 0.00  0.00 |
